# A mini review of current studies on metal-organic frameworks-incorporated composite solid polymer electrolytes in all-solid-state lithium batteries

**DOI:** 10.1016/j.heliyon.2023.e19746

**Published:** 2023-09-07

**Authors:** Phuoc-Anh Le, Nghia Trong Nguyen, Phi Long Nguyen, Thi Viet Bac Phung, Cuong Danh Do

**Affiliations:** a Center for Environmental Intelligence and College of Engineering and Computer Science, VinUniversity, Hanoi, 100000, Viet Nam; b Institute of Sustainability Science, Vietnam Japan University, Vietnam National University, Hanoi, 100000, Viet Nam; c School of Chemical Engineering, Hanoi University of Science and Technology, Hanoi, 100000, Viet Nam

**Keywords:** All-solid-state lithium batteries, Metal-organic frameworks, Solid polymer electrolytes, Electrochemistry, Energy storage

## Abstract

All-solid-state lithium batteries (ASSLBs) using solid polymer electrolytes (SPEs) are believed to be future next-generation batteries aiming to replace high-risk traditional batteries using liquid electrolytes, which have a wide application range in portable electronic devices, portable power supplies, and especially in electric vehicles. Moreover, the appearance of SPEs can overcome the electrolyte leakage and flammability problems in conventional lithium-ion batteries. Nevertheless, ASSLBs still face some limitations due to the low ionic conductivity of solid-state electrolytes (SSEs) at room temperature and the poor contact electrode/electrolyte interface, which can be solved by suitable strategies. Currently, the research strategies of metal-organic frameworks that can be incorporated into solid polymer electrolytes offer a remarkable method for producing uniform solid polymer electrolytes that have good electrode/electrolyte contact interfaces and high ionic conductivity. Herein, the updates of current studies about metal-organic framework-incorporated composite solid polymer electrolytes are discussed in this mini-review.

## Graphical abstract

**Figure:**
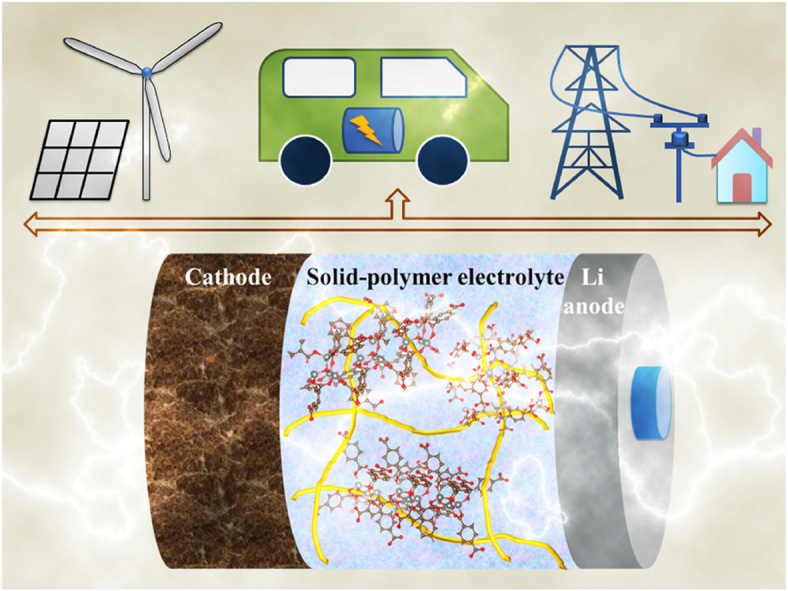

## 1 Introduction

The current limitations of natural sources and global warming caused by fossil fuels, particularly gas and coal, are pressing reasons for developing new renewable energy systems and energy storage. Many nations are currently concentrating on the development of electric transportation, corresponding to trains, buses, cars, and bikes [1,2]. These vehicles will integrate the latest sensor and artificial intelligence technologies to meet the requirements of rapid information processing. They have incorporated various contemporary technologies, producing larger amounts of energy that exceed the capacity of present batteries. Therefore, it is essential to create the following generation of highly energy-dense batteries with a long lifetime, especially those that are non-flammable due to chemical leakage and short circuits. The rapid advancement of electronic technology in the early 21st century has prompted the exploration of various solutions to optimize energy storage systems, particularly in the realm of batteries. One such solution is the implementation of solid-state lithium batteries, which rely on solid polymer electrolytes to address the issues of electrolyte leakage and the growth of lithium dendrites [3,4].

Since the first time they appeared in commercial production in 1991, lithium-ion batteries have become the most popular battery type [5,6]. However, there are several limitations in thermal stability and a volatile liquid electrolyte that prevent large cell fabrication. In order to address the challenges posed by traditional lithium-ion batteries, the introduction of ASSLBs is considered as a promising future-oriented battery technology that utilizes solely solid-phase components [7,8]. In ASSLBs, solid polymer electrolytes are the key point leading to thermal stability, lifetime, and capability of devices, which have many advantages including easy synthesis, cost-effective fabrication, large-scale fabrication [9,10]. In general, there are two primary categories of solid polymer electrolytes: inorganic solid-state electrolytes and organic solid-state electrolytes. Both of them show many unique attractive properties; inorganic solid electrolytes have high ionic conductivity, strong mechanical ability, and chemical stability [11], while organic solid electrolytes (also called solid polymer electrolytes) show flexible properties and are easy to make in larger sizes and thin films [12]. Nevertheless, they also have some disadvantages, such as poor contact electrode/electrolyte interface for inorganic solid electrolytes and low ionic conductivity for organic solid electrolytes [[13], [14], [15]]. Inorganic-organic hybrid solid polymer electrolytes have recently been created using fantastic methods to get over the aforementioned constraints. Among various electrolyte types, metal-organic frameworks (MOFs) are rising as a promising new candidate with more attraction due to their large surface area, ease of modification, and homogenous form with polymer chains [16]. Recently, there has been a significant focus on the development of ASSLBs utilizing solid polymer electrolytes, with the aim of creating next-generation devices that exhibit robust thermal stability and high energy density suitable for future electric vehicles.

MOFs, also called porous coordination polymers, are inorganic-organic composite materials made by the combination of metal ion-cluster complexes with multifunctional organic ligands as linkers [17]. Currently, more than twenty thousand MOFs have been developed with ultra-high specific surface areas based on micro- and meso-porous structures, which are frequently applied in various fields of sensing, catalysts, optoelectronics, drug delivery, and especially in energy storage and conversion like supercapacitors, batteries, and solar cells [[18], [19], [20], [21], [22]]. One of the most interesting properties of MOFs and composites is the amazing topology of the framework, which can be controlled by selecting the organic ligands at various lengths and group functions [19]. However, not all MOFs are suitable for use as electrolyte booster fillers in batteries. Depending on whether the physical, chemical, and electrical properties of each MOF type can meet the requirements for batteries or not, they will be studied through simulation methods to optimally support experiments. In addition, depending on the requirements of each type of electrolyte for solid-state lithium batteries, specific MOFs are synthesized for each requirement. Thus, we can approach the problem from two directions: (i) directly using MOF types for testing and fabrication on a laboratory scale; and (ii) depending on specific requirements, focusing on matching the MOFs accordingly. Thus, this variety of customization makes MOFs a great idea for incorporation into solid polymer electrolytes to create excellent uniform composites with many outstanding properties, including enhanced ionic conductivity, ionic mobility, and ion transfer numbers [20].

Based on many previous successes in research on enhancing battery performance based on incorporating MOF materials in the electrolyte system, functional MOFs and composites have recently been tested in solid-state batteries, which have shown positive results, especially when using MOFs mixed into polymer electrolyte to create pathways for lithium ions and also a buffer zone preventing lithium dendrite growth [23,24]. Due to their large surface area, adaptable porous structure, and extensive porousness, MOFs are ideal structures to study ionic conduction mechanisms and structure-property relationships. They also provide excellent chances for modifying the physicochemical and electrochemical behaviors of SSEs in ASSLBs. Moreover, MOFs are porous polymers with coordination, where organic ligands connect the metal centers or metal clusters to one another. The properties of the material can be modified by carefully choosing the organic connection and the metallic ion. As a result, it is feasible to regulate the porosity, size, three-dimensional structure, and distribution of the pores. MOFs provide a viable method for creating solid-state electrolytes for ASSLBs because of their high ionic conductivity, clear pore structure, and adjustable surface polarities. Recent work has demonstrated that a solid-state electrolyte modified with a MOF may manage the ion transport for homogenous lithium electrodeposition at a high current density, which could successfully suppress the lithium dendrite formation and provide a solution to the safety issues.

ASSLBs currently have some drawbacks, and the combination of MOFs and SPEs offers a potentially effective solution. MOF-SPEs can be divided into three groups based on the structural and morphological characteristics of the MOFs: (1) nanofiller-MOFs, (2) laden-MOFs, and (3) network-MOFs-incorporated composite solid polymer electrolytes. This mini-review provides an in-depth discussion of each group, along with the current research findings in ASSLB studies.

## 2 Metal-organic frameworks-incorporated composite solid polymer electrolytes

**Fig. 1:**
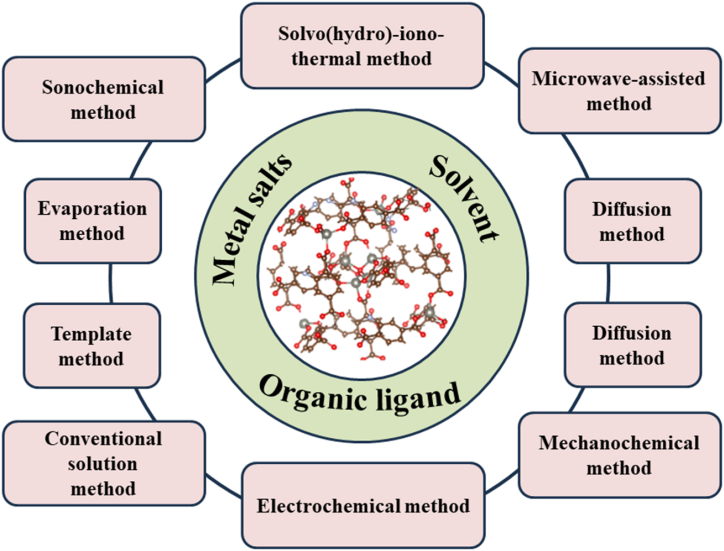
The methods to synthesize MOF materials.

The synthesis of MOFs is influenced by a number of variables, including reaction time, temperature, solvents, the type of metal ions and organic ligands, the size of the nodes and their structural, the presence of counterions, and the kinetics of crystallization that satisfy the target MOF requirements of porosity, morphology, and crystallinity. Fig. 1 shows various strategies to synthesize MOFs which can be utilized, depending on the resulting frameworks and properties [[17], [18], [19], [20], [21], [22], [23], [24]].

**Fig. 2:**
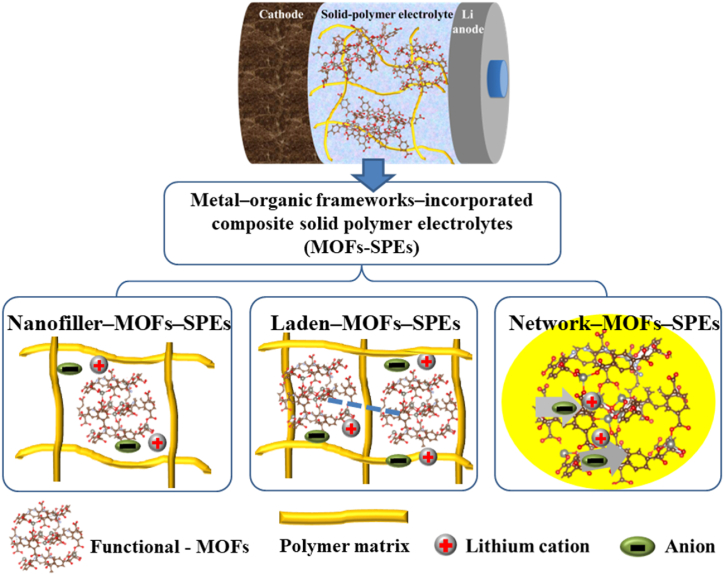
Classification of metal–organic frameworks–incorporated composite solid polymer electrolytes following by nanofillers-, laden-, and network-MOFs-GPEs.

Solid-state polymer electrolytes are the excellent candidate for all solid-state lithium batteries but they have some disadvantages such as their fragility and weak chemical stability under processing conditions. By gaining access to ion mobility from interactions with chemicals, solvent incopporation, and a freely flowing channel, MOF incopporation in a solid-state gel polymer system has the potential to enhance ionic conductivity. Based on their morphology and structures, MOFs can be mixed as nanofiller in a polymer matrix or can be used to bond with the polymer matrix, creating a new stable composite network [25,26]. Because of their flexible properties, MOFs are very useful for improving solid-state electrolytes in order to optimize the structure and scale up ASSLBs [[27], [28], [29], [30]]. Following, the three major categories of MOFs incorporating composite solid polymer electrolytes are discussed: (1) nanofiller-MOFs-SPEs, (2) laden-MOFs-SPEs, and (3) network-MOFs-SPEs (Fig. 2). Herein, the MOFs are often functionalized prior to incorporation, depending on the specifics of the polymer matrix.

### 2.1 Nanofiller–MOFs–incorporated composite solid polymer electrolytes

**Fig. 3:**
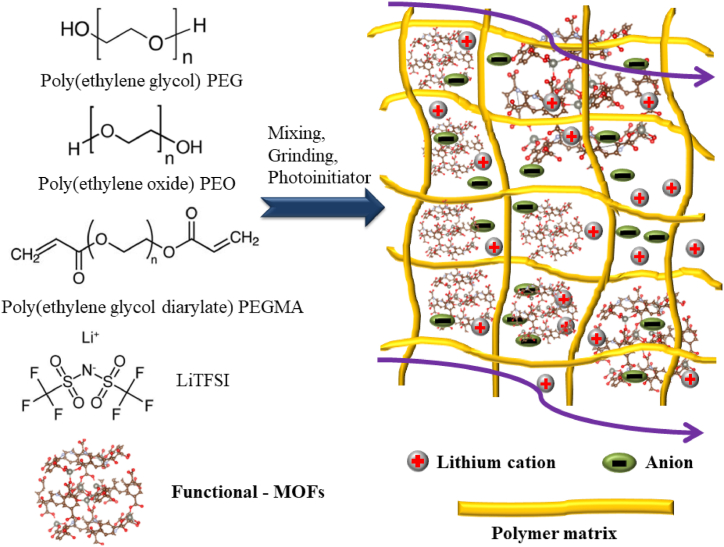
General process to synthesize nanofiller–MOFs–SPEs.

In order to create nanofiller MOF-SPEs, MOFs are blended directly into polymer networks, occasionally with the addition of liquid plasticizers and metal salts (Fig. 3). Typically, the incorporation of micro- and nanoparticles MOFs into polymer chains as the fillers obtaining the inorganic-organic network provides additional transport channels for lithium ions and optimizes the interface contact electrode/electrolyte, which improves not only ionic conductivity but also enhances electrochemical stability. Herein, the enhanced ion mobility and migration pathways help to increase ionic conductivity [31,32]. Angulakshmi et al. reported UiO-66-NH_2_@SiO_2,_ which was synthesized and dispersed in a polymer electrolyte solution of PEO-LiTFSI before casting and drying to obtain a solid-state polymer electrolyte. In this report, the MOFs-GPEs with UiO-66-NH_2_@SiO_2_ filler obtained high ionic conductivity of 8.1 × 10^−6^ S cm^−1^ and high specific capability of 119 mAh g^−1^ at 60 °C [33]. Following the modification of the MOFs method, Zhang et al. reported the photoinitiator benzophenone (BP) and HKUST-1 (also called MOF-199) supported PEO polymer matrix to obtain a solid electrolyte (UV-PEO-HKUST-1) with a high ionic conductivity of 4.99 × 10^−3^ S cm^−1^ at 80 °C and a solid-state battery Li/UV–PEO-HKUST-1/LiFePO_4_ obtained a good specific capability of 158.8 mAh g^−1^ at 0.1 C [34]. Based on previous research, Liu et al. introduced a polycaprolactone (PCL) modified UiO-66-NH_2_ mixing with boronic ester crosslink monomer (BEM) and poly (ethylene glycol) diacrylate (PEGDA) with the presence of LiTFSI under the name BPM-CSPE [35]. At 80 °C, this solid electrolyte system achieved an excellent ionic conductivity of 1.04 × 10^−4^ S cm^−1^ with 30% addition of LiTFSI, that is greater than the situation without PCL-MOFs (only 2.82 × 10^−5^ S cm^−1^) and Li/BPM-CSPE/LiFePO_4_ showed a good capacity for discharge of 145.5 mAh g^−1^ at 60 °C. A recently developed all-solid-state composite electrolyte for lithium-ion batteries with outstanding durability incorporates MOF-5, as presented by Wen and coworkers [36]. Additionally, in an effort to increase the polymer matrix’s mechanical and thermal durability, their excellent idea of random copolymerization was prepared by random polymerization of triﬂuoroethyl methacrylate (TFEMA) and poly(ethylene glycol) methacrylate (PEGMA) to obtain P(TFEMA-ran-PEGMA) polymer. They optimized the solid-state electrolyte system by mixing MOF-5 particles and LiTFSI into P(TFEMA-ran-PEGMA) system [36]. Following their report, MOF-5/LiTFSI/TFEMA/PEGMA exhibited a high value of approximately 10^−5^ S cm^−1^ at 30 °C and increased to 0.51 × 10^−5^ S cm^−1^ at 60 °C, and the LiFePO_4_/MOF-5 modified solid-state electrolyte/Li obtained a specified capacity for discharge of 116 mAh g^−1^ which is better than without MOF-5 (91 mAh g^−1^). Following MOFs studies, Chen and coworkers introduced MOF Zif-67 nanoparticles incorporated in [Py13][TFSI] with LiTFSI electrolyte systems to form ILE@MOF with the maximum ionic conductivity of 2.29 × 10^−3^ S cm^−1^ at 30 °C, and the Li/ILE@MOF/LiNi_0.33_Mn_0.33_Co_0.33_O_2_ cells obtained a good discharge specific capability of 147.3 mAh g^−1^ at 120 °C [37]. One idea of metal functional MOFs to obtain 3D porous materials was introduced by Wu et al. when they prepared 3D-Ce-UiO-66 ([Ce_6_O_4_(OH)_4_(BDC)_6_]) as nanofiller and mixed it in PEO–LiTFSI [38]. Their nanofiller–MOFs–GPEs with 10 wt% depicted the greatest ionic conductivity of 3 × 10^−5^ S cm^−1^ at 30 °C and 3.1 × 10^−4^ S cm^−1^ at 60 °C, respectively, which is higher than only PEO-LiTFSI (6.7 × 10^−6^ S cm^−1^ at 30 °C and 2 × 10^−4^ S cm^−1^ at 60 °C at the same condition, respectively). These results suggest that the incorporation of 3D Ce-UiO-66 composites provides abundant cavities, which improve ion mobility and increase ionic conductivity by allowing greater Lewis acid-base interactions between oxygen in polymer PEO and lithium ions in salt [38]. Following, their battery cells: Li/(3D-Ce–UiO-66–LiTFSI–LEO)/FeF_3_ exhibited a maximum discharge specific capability of 300 mAh g^−1^ after 230 cycles at 0.1C under 60 °C while Li/(3D-Ce–UiO-66–LiTFSI–LEO)/LiFePO_4_ obtained a good value of 120 mAh g^−1^ at 0.5C under 60 °C.

**Fig. 4:**
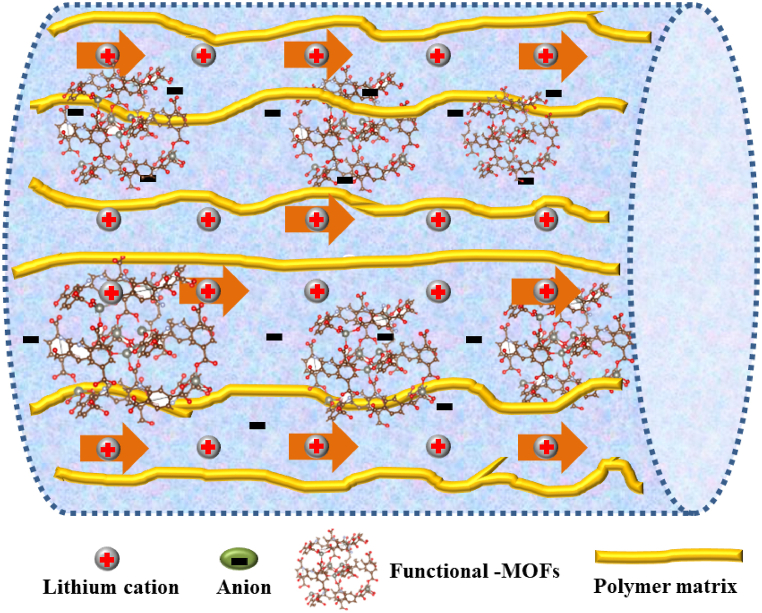
The standard ion transport model of nanofiller–MOFs–SPEs system [[33], [34], [35], [36], [37]].

The above results suggest that adding MOFs to polymer electrolyte matrix could improve intermolecular interactions between MOF filler and polymer-network which improves the dissociation of lithium ions (Table 1). Herein, the polymer network is considered a conduit with MOF filler inside that promotes lithium cation migration and also enhances the surface contact between the electrolyte and the lithium metal anode (Fig. 4).

**Table 1:** Table listing the various MOF types included in the solid polymer electrolyte used in solid-state lithium batteries.

Structure	Ionic conductivity	Anodic steady window (V)	Batteries types Anode/cathode & performance	Ref. & year
Nanofiller-MOFs-SPEs				
UiO-66-NH_2_@SiO_2_ + PEO/LiTFSI	8.1 × 10^−6^ S cm^−1^ at 60 °C	–	Li/LiFePO_4_ 119 mAh g^−1^ at 60 °C	33 (2020)
photoinitiator BP-HKUST-1-PEO	4.99 × 10^−3^ S cm^−1^ at 80 °C	5.25	Li/LiFePO_4_ 158.8 mAh g^−1^ at 0.1C	34 (2021)
polycaprolactone (PCL) modiﬁed UiO-66-NH_2_ + poly (ethylene glycol) diacrylate (PEGDA)/LiTFSI	1.04 × 10^−4^ S cm^−1^ at 80 °C	5.29	Li/LiFePO_4_ 145.5 mAh g^−1^ at 60 °C	35 (2021)
MOF-5/LiTFSI + TFEMA/PEGMA	0.51 × 10^−5^ S cm^−1^ at 60 °C	5.38	Li/LiFePO_4_ 116 mAh g^−1^ at 60 °C	36 (2021)
Zif-67 + [Py13][TFSI]/LiTFSI	2.29 × 10^−3^ S cm^−1^ at 30 °C	5.4	Li/LiNi_0.33_Mn_0.33_Co_0.33_O_2_ 147.3 mAh g^−1^ at 120 °C	37 (2019)
3D-Ce-UiO-66 + PEO/LiTFSI	3 × 10^−5^ S cm^−1^ at 30 °C & 3.1 × 10^−4^ S cm^−1^ at 60 °C	4.5	Li/FeF_3_: 300 mAh g^−1^ at 0.1C under 60 °C Li/LiFePO_4_: 120 mAh g^−1^ at 0.5C under 60 °C	38 (2021)
**Laden-MOFs-SPEs**				
Ni_3_-(BTC)_2_-MOF + PEO/LiTFSI	1.4 × 10^−4^ S cm^−1^ at 30 °C and 4.5 × 10^−3^ S cm^−1^ at 70 °C	–	Li/LiFePO_4_ 127 mAh g^−1^ at 0.05C	39 (2016)
Al-TPA-MOF + PEO/LiTFSI	0.1 mS cm^−1^ at 60 °C	–	Li/LiFePO_4_ 130 mAh g^−1^ after 100 cycles at 0.1C	40 (2018)
UiO-66-NH_2_ + PEO/LiTFSI	3.56 × 10^−4^ S cm^−1^ at 60 °C	4.5	Li/LiFePO_4_ 164 mAh g^−1^	41 (2020)
Ni_2_(OH)_2_-BDC + PEO/LiTFSI	1.66 × 10^−5^ S cm^−1^ at 25 °C	4.9	Li/LiFePO_4_ 130 mAh g^−1^ after 50 cycles at 0.1C	42 (2020)
**Network-MOFs-SPEs**				
**(i) MOFs linking polymer matrix**				
Trimethylamine modified UiO-66-NH_2_ + PEGDA	4.31 × 10^−5^ S cm^−1^ at 30 °C	5.5	Li/LiFePO_4_ 151 mAh g^−1^ at 60 °C	43 (2018)
D-UiO-66-NH_2_ + PEO/LiTFSI	3.1 × 10^−5^ S cm^−1^ at 25 °C & 6.3 × 10^−4^ S cm^−1^ at 60 °C	5	Li/LiFePO_4_ 126.4 mAh g^−1^ at 60 °C	44 (2019)
10-HKUST-1 + PEO/LiTFSI	3.5 × 10^−4^ S cm^−1^ at 50 °C and 2.4 × 10^−3^ S cm^−1^ at 80 °C	4.71	Li/LiFePO_4_ 158 mAh g^−1^ at 0.1C under 60 °C	45 (2021)
**(ii) MOFs-polymer filler**				
UiO-66/Li-Il + PEO/LiTFSI	1.7 × 10^−3^ S cm^−1^ at 30 °C	–	Li/LiFePO_4_ 151 mAh g^−1^ at a rate of 0.5C of 60 °C	46 (2019)
UiO-66/(LiPF_6_/EC-DMC-DEC) + PEO/LiTFSI	1.47 × 10^−4^ S cm^−1^ at 30 °C, and 9.63 × 10^−4^ S cm^−1^ at 60 °C	5.2	Li/LiFePO_4_ 137.9 mAh g^−1^ at a rate of 0.1C of 60 °C	47 (2020)
Lithiated Cu-MOF-74 + PEO/LiTFSI	5.5 × 10^−5^ S cm^−1^ at 30 °C and 1.3 × 10^−3^ S cm^−1^ at 80 °C	4.8	Li/LiFePO_4_ 152 mAh g^−1^ at a rate of 0.1C of 60 °C	48 (2022)

From this point of view, the nanofiller–MOFs–SPEs are easily prepared, but it is also difficult to control the MOFs distribution in the polymer electrolyte matrix, which cannot optimize the maximum ionic conductivity and reduce the interface resistance of the layer between solid-state electrolytes and lithium metal anodes. Therefore, it is imperative to optimize this approach to achieve a homogeneous gel polymer electrolyte before the casting and drying processes in order to create SSEs.

### 2.2 Laden–MOFs–GPEs

**Fig. 5:**
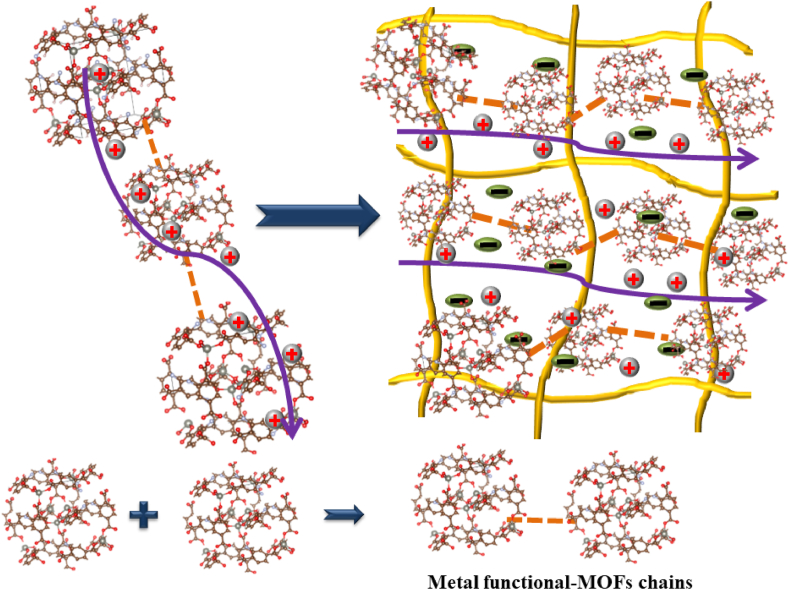
The standard ion transport model of laden–MOFs–SPEs system [[39], [40], [41], [42]].

Aiming to improve MOFs-SPEs ion conduction, laden-MOF structures have been created by linking multiple chains of functional MOFs together to form a long chain (Fig. 5). These structures are expected to form a conducting channel, improving the ion’s mobility. It makes sense to use metal functional groups to increase the conductivity of MOFs. For example, a nickel-1,3,5-benzene tricarboxylate metal-organic framework (Ni_3_–(BTC)_2_–MOF) laden was synthesized and mixed in gel polymer electrolyte of PEO/LiTFSI to obtain a Ni_3_-(BTC)_2_-MOF–PEO/LiTFSI solid polymer electrolyte system [39]. This structure (Ni_3_-(BTC)_2_-MOF 10 wt%, LiTFSI 15 wt%, and PEO 70 wt%) exhibited the highest ionic conductivity of 1.4 × 10^−4^ S cm^−1^ at 30 °C and 4.5 × 10^−3^ S cm^−1^ at 70 °C, respectively. The battery cell Li/[Ni_3_-(BTC)_2_-MOF–PEO/LiTFSI]/LiFePO_4_ obtained a good specified capacity for discharge of 127 mAh g^−1^ at 0.05C [39]. Following the above idea, an aluminium terephthalic acid metal-organic framework (Al-TPA-MOF)-laden composite was prepared for ASSLBs [40]. This structure showed excellent thermal stability up to 270 °C and very high ionic conductivity of 0.1 mS cm^−1^ at 60 °C (with PEO/Al-TPA-MOF/LiTFSI in 80:10:10 wt%). The ASSLBs cell of Li/(PEO/Al-TPA-MOF/LiTFSI)/LiFePO_4_ delivered 130 mAh g^−1^ after 100 cycles at 0.1C. Herein, the high concentration of up to 10 wt% of Al-TPA-MOF nanofiller improves the amorphous content of polymer matrix PEO through the interaction of Al-TPA-MOF with PEO, and PEO with lithium ions [40]. Recently, Qing et al. reported an amine-functional UiO-66 laden incorporated in PEO/LiTFSI as a solid-state electrolyte for stable ASSLBs [41]. Their study focused on the preparation of UiO-66-NH_2_ laden with chain structure, which enhances lithium ion mobility through polar UiO-66-NH_2_ bridges and prevents the agglomeration of lithium salt in the PEO matrix. Thus, the UiO-66-NH_2_-PEO/LiTFSI system obtained 3.56 × 10^−4^ S cm^−1^ at 60 °C and increased up to 9.74 × 10^−4^ S cm^−1^ at 80 °C which is higher than only PEO/LiTFSI with 3.3 × 10^−4^ S cm^−1^ at 60 °C. The battery cell based on MOFs-SPEs structure: Li/(UiO-66-NH_2_-PEO/LiTFSI)/LiFePO_4_ illustrated a good specified capacity for discharge of 164 mAh g^−1^ [41]. It can be seen that UiO-66-NH_2_ with its chain structure serves well as the laden component in the PEO/LiTFSI polymer system. Further, aim to enhance MOF conductivity, the two-dimensional (2D) structure of functional-MOFs is a novel and good strategy that has more attractions. Han et al. developed 2D nickel-based ultrathin MOF nanosheets [Ni_2_(OH)_2_-benzene dicarboxylic acid (BDC)], which were mixed with PEO/LiTFSI in order to enhance the amorphous phase of PEO and thus improve the ionic conductivity of SPEs [42]. Moreover, the 2D chain of functional-MOF with Ni(OH)_2_ could promote the Lewis acid–base between nickel atoms and lithium ions, which increases the lithium salts' dissociation. The ionic conductivity of the Ni_2_(OH)_2_-BDC-PEO/LiTFSI was 1.66 × 10^−5^ S cm^−1^ at 25 °C. The all-solid-state Li metal battery cell Li/(Ni_2_(OH)_2_-BDC-PEO/LiTFSI)/LiFePO_4_ exhibited a discharge specific capacity of 130 mAh g^−1^ after 50 cycles at 0.1C at 30 °C [42].

It can be suggested that the metal-functional MOFs making long chains that are integrated into the matrix of polymer electrolytes not only help to uniformly distribute lithium ions but also enhance Lewis acid-base between functional MOF atoms and lithium ions (Table 1).

### 2.3 Network-MOFs-SPEs

**Fig. 6:**
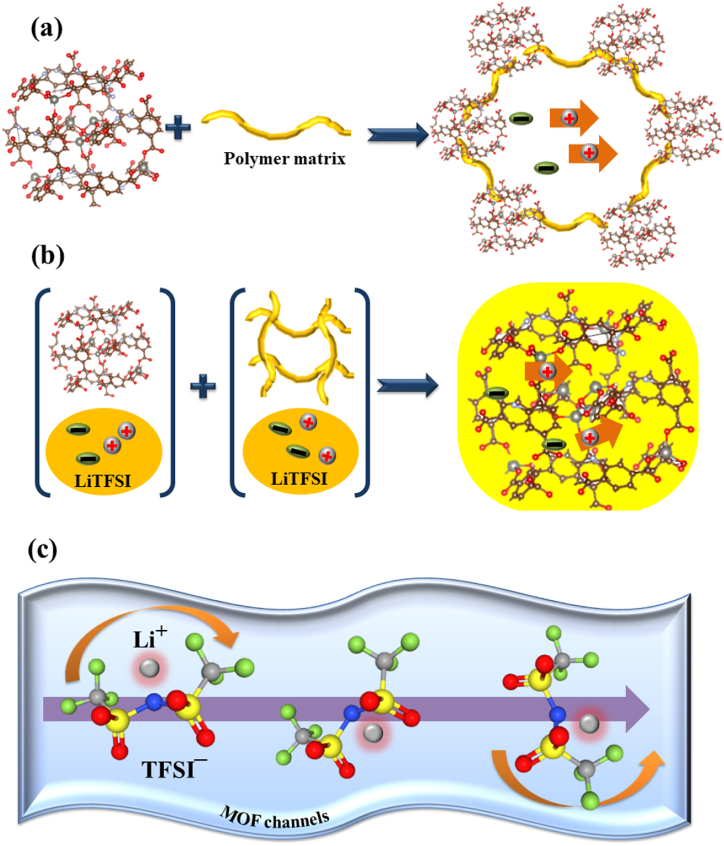
The standard ion transport model of network–MOFs–SPEs system for (a) MOFs linking polymer matrix, (b) MOFs host frame, and (c) electrolyte ion transport imposed by the “caged MOFs” [[43], [44], [45], [46], [47], [48]].

Network MOFs-SPEs, also called sidechain MOFs, have been developed to enhance the ionic conductivity, electrolyte/electrode interface, and especially the thermal stability of SSEs. Herein, the crosslinking method aims to immobilize MOFs in the polymer matrix as one of the components that make up the additional transport pathway for lithium ions. There are two types of network MOF-SPEs: the first type uses MOFs linking polymer matrix to immobilize MOF chains, and the second type uses MOFs as hosts for filling by gel polymers (Fig. 6).

Recently, Wang et al. reported trimethylamine modified UiO-66-NH_2_ MOFs nanoparticles to obtain M-UiO-66-NH_2_, mixing into [poly(ethylene glycol) diacrylate (PEGDA), LiTFSI] system under the photoinitiator to form a square topology of M-UiO-66-NH_2_-PEGDA by a covalent link where UiO-66-NH_2_ is the link and PEGDA is the interconnection [43]. The maximum ionic conductivity of 4.31 × 10^−5^ S cm^−1^ at 30 °C was demonstrated by the structure’s best weight ratio of M-UiO-66-NH_2_ and PEGDA (1:8, HSPE-1-8) and the cell with sandwich structure: LiFePO_4_/HSPE-1-8/Li obtained an average discharge capacity of 151 mAh g^−1^ at 60 °C. Another report of functional-UiO66 was introduced: D-UiO-66-NH_2_ (CMOF) [44]. After a simple step of mixing and grinding dried CMOF and PEO/LiTFSI following hot pressing, a uniform P@CMOF composite membrane around 40 μm was obtained with the immobility of CMOF in the matrix. The electrochemical characterizations of P@CMOF illustrated good results for the highest ionic conductivity of 3.1 × 10^−5^ S cm^−1^ at 25 °C for adding 12.5 vol% of CMOF and up to 6.3 × 10^−4^ S cm^−1^ at 60 °C, and the solid-state battery cell of Li/P@CMOF/LiFePO_4_ delivered the maximum discharge capacity of 126.4 mAh g^−1^ at 0.5 C [44]. This approach offers a cheap and ecologically beneficial solution. Moreover, the combination between MOF and polymer matrix not only allows the lithium ions in the new combination matrix but also enhances the conduction of interface polymer/MOF (Fig. 6a). Another innovative concept was the use of 10-HKUST-1 MOF as a multipurpose addition to alter solid polymer electrolytes [45]. The 10-10-HKUST-1 functional PEO/LiTFSI has a high ionic conductivity of 3.5 × 10^−4^ S cm^−1^ at 50 °C and 2.4 × 10^−3^ S cm^−1^ at 80 °C. The high ionic conductivity of 10-HKUST-1/PEO/LiTFSI due to the addition of 10-HKUST-1 can capture the anions in TFSI^–^ and also increase the amorphous phase, enhancing the ion’s mobility. The battery cells: LiFePO_4_/(10-HKUST-1/PEO/LiTFSI)/Li exhibited the discharge specific capacity of 158 mAh g^−1^ at 0.1 C.

In order to improve the composite structure of MOFs-SPEs, a new strategy is to create a MOF’s host, which is immersed in lithium ionic liquid and filled with polymer to obtain a stable MOFs-SPEs system (Fig. 6b). This method provides a promising porous structure that can not only capture the anions in caged MOFs but also reduce lithium dendrite. Following this principle, Wu and Gou provided the idea of using a porous MOFs of UiO-66 as an assistant ionic conductor when impregnating UiO-66 in (LiTFSI + [EMIM][TFSI], named Li-IL in short) ionic liquid electrolyte to obtain UiO-66/Li-IL filler [46]. This composite host was filled by PEO/LiTFSI following casting and drying to obtain MOFs-SPEs membranes: PEO-n-UiO. With 20% UIO/Li-IL, the PEO-n-UiO showed a high value for ionic conductivity of 1.7 × 10^−3^ S cm^−1^ at 30 °C. The ASSLBs cell following structure the LiFePO_4_/PEO-n-UiO/Li obtained a specific discharge capacity of 151 mAh g^−1^ at a rate of 0.5C at 60 °C [46]. One other study of UiO-66 as an impregnating host was reported by Zhang and coworkers [47]. They immersed the UiO-66 framework in an ionic liquid electrolyte of LiPF_6_/EC-DMC-DEC to obtain the UiO-66/IEs filler. Then, the mixture of UiO-66/IEs with PEO/LiTFSI was prepared, cast, and dried to obtain a solid-state membrane electrolyte that displayed a high ionic conductivity of 1.47 × 10^−4^ S cm^−1^ at 30 °C, and rose to 9.63 × 10^−4^ S cm^−1^ at 60 °C, leading the Li/(UiO-66/IEs-PEO/LiFSI)/LiFePO_4_ cell to obtain a good discharge specific capacity of 137.9 mAh g^−1^ at a rate of 0.1C at 60 °C [47]. Continuously, the following MOF host type, the MOF-SPEs membranes using Cu-MOF-74, was introduced [48]. Firstly, Cu-MOF-74 was synthesized by the solvothermal method and immersed in LiTFSI to obtain Lithiated Cu-MOF-74 particles. Then, this Lithiated Cu-MOF-74 was filled by PEO/LiTFSI, followed by casting and during to obtain solid composite polymer electrolyte membranes, named Li-MOF/PEO in short. The electrochemical characterization of the Li-MOF/PEO membrane with the addition of 40 wt% Lithiated Cu-MOF-74 illustrated a good value of 5.5 × 10^−5^ S cm^−1^ at 30 °C and an increase to 1.3 × 10^−3^ S cm^−1^ at 80 °C, leading the Li/(Li-MOF/PEO)/LiFePO_4_ cell to maintain a high discharge capacity value of 152 mAh g^−1^ after 300 cycles at 0.1 C under 60 °C [48]. According to the studied findings, the network of MOFs-SPEs offers effective cages for enhancing the uniformity of ionic transport, as seen in the case of LiTFSI in Fig. 6c, for instance. The electrolyte is captured in the MOFs-SPEs composite network and can rapidly diffuse in the cages, which prevents the reaction of lithium ions with the lithium metal surface electrode, reducing the Li dendrite [[48], [49], [50]].

## 3 Mechanism study of MOFs-SPEs

Density functional theory (DFT) simulations can give insightful information on the MOFs-SPEs' properties and guide the optimization of their composition and structure for improved performance in ASSLBs. The first principles of DFT modeling techniques have grown in popularity for studying material structures and electrochemical reactions. DFT enables the calculation of atomic structural energies and the examination of ionic transport channels in SSEs. As a result, DFT has become a valuable tool for researchers studying the properties of SSEs and developing new materials for use in solid-state batteries. DFT is a widely used computational method that allows for the prediction and analysis of various materials' properties, including MOFs and SPEs. By simulating the electronic structure and interactions of the components of a MOFs-SPEs composite, DFT can provide insights into the mechanism of ionic conductivity enhancement and predict the material’s physical and chemical properties.

**Fig. 7:**
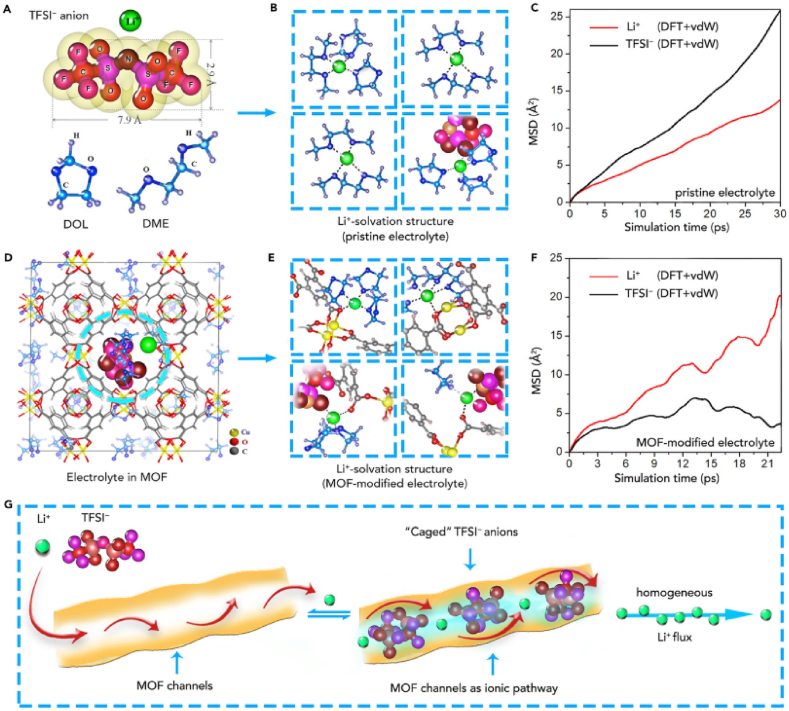
Molecular and crystal structures of LiTFSI in MOFs using MD simulation: (a) LiTFSI and DOL/DME solvent molecular structure, (b) LiTFSI@DOL/DME solvation structure electrolyte, (c) Li^+^ and TFSI ions' square displacement (MSD) in a pure electrolyte as a function of simulation duration, (d) LiTFSI@DOL/DME electrolyte is incorporated into the MOF host frame, (e) Structures of the MOF-modified electrolyte’s Li^+^ solvation, (f) Relationship between the MSD of Li+ and TFSI ions and simulation time in MOF-modified electrolytes, (g) the transportation of LiTFSI in the MOF host. Fig. 7 Reproduced with permission from ref 54. Copyright 2018 Elsevier.

Several studies have employed DFT simulations to study the ionic transport and electronic properties of MOFs-SPEs [51,52]. For example, Kihun et al. presented a new lithium sulfonated covalent organic framework (TpPa-SO_3_Li) solid-state electrolyte [53]. DFT calculations are used to study the theoretical explanation of lithium ion conduction behavior in TpPaSO_3_Li. As shown in the energy diagrams, initial (IS), intermediate (IM), transition (TS), and final (FS) computations are used to determine the migration barriers (Em) of lithium ions in both axial and planar routes. Using practical investigations, density functional theory computations, and molecular dynamics simulations, Songyan and colleagues presented a method of MOF (HKUST-1) modified electrolyte to accomplish selective ion transport [Fig. 7a–f]. In this procedure, the ''caged'' electrolyte anions (TFSI-) in the angstrom-scale pores of MOF could aid in a consistent Li ion flow (Fig. 7g) [54]. With a substantially greater current density of roughly 7 mA cm^−2^, the resultant MOF-modified electrolyte demonstrated steady performance through 2000 cycles at 5 °C.

**Fig. 8:**
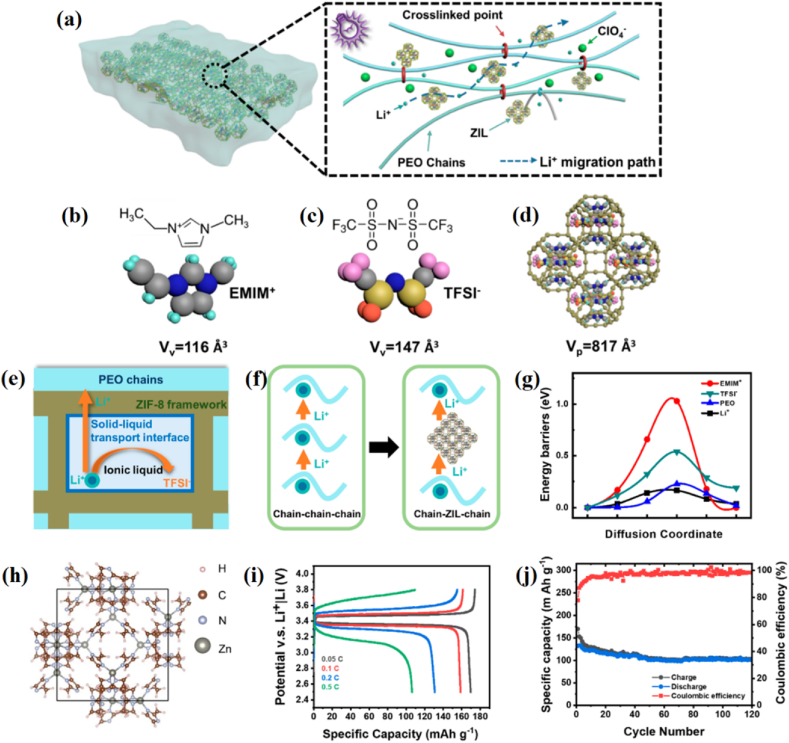
ASSLBs simulation and experiment studies using solid-polymer electrolyte system of ZIF-8:(EMIM_0.83_Li_0.17_)TFSI: (a) A schematic representation of the UV cross-linked composite solid electrolyte and the migration channel for Li ions, (b) van der Waals volumes of EMIM^+^ (c) van der Waals volumes of TFSI^−^, (d) The structure of ZIF- incorporating into (EMIM_0.83_Li_0.17_)TFSI, (e) scheme of Li ion transport process at a solid–liquid interface, (f) structured conﬁguration of ZIF-8, (i) LiFePO_4_|C−CSE_3_| lithium battery performance while charging and discharging at various current rates of 0.05, 0.1, 0.2, and 0.5 C, (f) LiFePO_4_|C−CSE_3_| lithium battery cycling stability at 0.1 C charge/discharge rate. Fig. 8 reprints with permission from Ref. 55 copyright 2020 American Chemical Society.

With the ambition to enhance ion transport parth at the interface electrode/electrolyte, Xia et al. presented a strategy to incorporate ZIF-8 into solid polymer electrolyte (Fig. 8). Here, poly(ethylene oxide) (PEO) and the ionic conductor ZIF-8 were used to construct a novel ultraviolet (UV) cross-linked composite solid electrolyte. The simulation of combination of ZIF-8, bis-(triﬂuoromethane)sulfonimide lithium salt (LiTFSI), and ionic liquid 1-ethyl-3-methylimidazolium-bis(triﬂuorome-thylsulfonyl)imide (EMIM-TFSI), and PEO was studied [55]. To build a solid-liquid transport interface between polymer chains and ZIF-8 in C-CSE, the porous ZIF-8 hosts act as stable 3D frameworks to absorb (EMIM_0.83_Li_0.17_)TFSI and restrain the movement of EMIM^+^ and TFSI^−^ [Fig. 8b–h]. They found that by promoting continuous ion transportation and increasing amorphous area, UV irradiation can reduce polymer crystallization and thus boost ionic conductivity [55]. Moreover, their MOFs-PEO-LiTFSI-EMIM-TFSI structure provides an excellent ion path for Li ions, which can move through MOF structures (Fig. 8a). In this study, the date of simulation and experiment illustrates the good results of lithium batteries with high performance and long life cycles (Fig. 8i and j), which indicate the usefulness of the MOF ZIF-8 serving as a connected way for ion transportation.

The studies used the DFT calculation method to theoretically explain the various experimental results, such as geometrical properties, electrical characteristics, and physical characteristics. This method also helped clarify the migration mechanism of ions in the mixed medium of MOFs and solid polymer host frames. It was done by creating a replica of the region of interest with the proper conditions. In situations where testing is not available or impossible at that time, DFT can also be used to simulate novel potential materials and understand the behavior of a particular regional material characteristic.

## 4 Challenges and future perspectives

The recently developed synthesis methods for functional-MOF materials provide a bright strategy to improve ASSLBs with cost-effectiveness. Moreover, the diversity of functional groups, metal species, and pore structure provides many choices for various types of solid-state electrolytes, which is a special advantage of MOF materials. Under perfect simulation conditions, ASSLBs can have three times higher energy densities by using a lithium metal anode, which increases the energy density of the composite cathode. Moreover, the solid-state polymer electrolytes are considered non-combustion or at least can prevent self-ignition, which reduces the risk of thermal runaway and allow the fabrication of large cell packaging.

However, ASSLBs are currently at a basic research level with high production costs and scalability limitations. Furthermore, the process of manufacturing solid electrolytes is still a major challenge, which is related to large-scale cell manufacture as well as the cost of the final product. Concerns about the stability of ASSLBs are raised by the usage of MOF components, electrochemical stability, and decomposition temperature, necessitating further investigation for future development. Improving the crystal size and specific surface area leads to an enhance porous structure, which allows more ion transportation.

(i) Integration of more functional polymers to broaden the diversity of MOFs-SPEs for different individual all-solid-state batteries.
(ii) The interactions between the many components inside the electrolyte, as well as those between solid electrolytes and electrodes, require extensive study, with a special focus on the interactions between MOFs and polymer matrices.
(iii) Functional-MOF materials can be used as fillers, laden, or network with polymer electrolyte to improve the ionic conductivity and contact interface electrolyte/electrode, but the detailed ion transportation mechanism needs to be studied deeply, aiming to explain clearly the structure and performance relationship.

## 5 Conclusions

The development of MOF materials as a crucial element in solid polymer electrolytes for all-solid-state lithium batteries is a possible step toward the next generation of batteries. With the advantages of diverse types, diversity of functional groups, and various different synthesis strategies, MOFs provide a new research direction for making composites with polymer electrolytes that aims to enhance the performance of ASSLBs through increasing ionic conductivity and improving the electrolyte/lithium metal electrode contact interface, corresponding to reducing the growth rate of lithium dendrites. The use of MOFs and functional-MOFs as one component in the solid-state polymer electrolyte for ASSLBs not only improves the thermal, mechanical, and flexible properties of SPEs but also improves the electrochemical stability and performance of batteries, which illustrate promising applications in energy storage applications. Therefore, in order to offer an overview and future possibilities, this mini-review gathers the most recent and innovative research on composite SPEs for ASSLBs that integrate MOF.

## Author contribution statement

All authors listed have significantly contributed to the development and the writing of this article.

## Declaration of competing interest

The authors declare that they have no known competing financial interests or personal relationships that could have appeared to influence the work reported in this paper

## Abbreviation

PEO: Poly(ethylene oxide)
LiTFSI: Lithium bis(trifluoromethanesulfonyl)imide, LiC₂F₆NO₄S₂
UiO-66: Zirconium 1, 4-dicarboxybenzene, C48H28O32Zr6 or Zr_6_O_4_(OH)_4_(OOC–C_6_H_4_–COO)_6_
HKUST-1 (or MOF-199): Cu_2_(CO_2_)_4_ cluster linked by trimesate
MOF-5 (or IRMOF-1): Zn_4_O(1,4-benzodicarboxylate)_3_ C_24_H_12_O_13_Zn_4_
[Py13][TFSI]: *N*-propyl-*N*-methylpyrrolidinium bis(trifluoromethylsulfonyl), C_10_H_18_F_6_N_2_O_4_S_2_
Zif-67: zeolitic imidazolate framework-67, or 2-methylimidazole cobalt salt, C_8_H_10_N_4_Co
EC-DMC-DEC: ethylene carbonate (EC)- dimethyl carbonate (DMC)- diethyl carbonate (DEC)
Cu-MOF-74: Cu_2_(2,5-dihydroxyterephthalate)(H_2_O)_2_·8H_2_O

## References

[1] Masias A., Marcicki J., Paxton W.A. (2021). Opportunities and challenges of lithium ion batteries in automotive applications. ACS Energy Lett..

[2] Sun Y.K. (2020). Promising all-solid-state batteries for future electric vehicles. ACS Energy Lett..

[3] Li C., Wang Z.Y., He Z.J., Li Y.J., Mao J., Dai K.H., Yan C., Zheng J.C. (2021). An advance review of solid-state battery: challenges, progress and prospects. SMT Trends.

[4] Zhao Q., Stalin S., Zhao C.Z., Archer L.A. (2020). Designing solid-state electrolytes for safe, energy-dense batteries. Nat. Rev. Mater..

[5] Ozawa K. (1994). Lithium-ion rechargeable batteries with LiCoO_2_ and carbon electrodes: the LiCoO_2_/C system. Solid State Ionics.

[6] Yoshino A. (2012). The birth of the lithium-ion battery. Angew. Chem. Int. Ed..

[7] Sarkar S., Thangadurai V. (2022). Critical current densities for high-performance all-solid-state Li-metal batteries: fundamentals, mechanisms, interfaces, materials, and applications. ACS Energy Lett..

[8] Lu Y., Zhao C.Z., Yuan H., Cheng X.B., Huang J.Q., Zhang Q. (2021). Critical current density in solid-state lithium metal batteries: mechanism, influences, and strategies. Adv. Funct. Mater..

[9] Manthiram A., Yu X., Wang S. (2017). Lithium battery chemistries enabled by solid-state electrolytes. Nat. Rev. Mater..

[10] Lu Y., Zhao C.Z., Yuan H., Cheng X.B., Huang J.Q., Zhang Q. (2021). Critical current density in solid-state lithium metal batteries: mechanism, influences, and strategies. Adv. Funct. Mater..

[11] Mishra A.K., Chaliyawala H.A., Patel R., Paneliya S., Vanpariya A., Patel P., Ray A., Pati R., Mukhopadhyay I. (2021). Review—inorganic solid state electrolytes: insights on current and future scope. J. Electrochem. Soc..

[12] Zhao L., Lakraychi A.E., Chen Z., Liang Y., Yao Y. (2021). Roadmap of solid-state lithium-organic batteries toward 500 Wh kg^-1^. ACS Energy Lett..

[13] Zhao Y., Wang L., Zhou Y., Liang Z., Tavajohi N., Li B., Li T. (2021). Solid polymer electrolytes with high conductivity and transference number of Li ions for Li-based rechargeable batteries. Adv. Sci..

[14] Yue L., Ma J., Zhang J., Zhao J., Dong S., Liu Z., Cui G., Chen L. (2016). All solid-state polymer electrolytes for high-performance lithium ion batteries. Energy Storage Mater..

[15] Hou T., Xu W. (2023). Deep dive into anionic metal-organic frameworks based quasi-solid-state electrolytes. J. Energy Chem..

[16] Fu X., Yu D., Zhou J., Li S., Gao X., Han Y., Qi P., Feng X., Wang B. (2016). Inorganic and organic hybrid solid electrolytes for lithium-ion batteries. CrystEngComm.

[17] Zhou H.C., Long J.R., Yaghi O.M. (2012). Introduction to metal–organic frameworks. Chem. Rev..

[18] Stock N., Biswas S. (2012). Synthesis of metal-organic frameworks (MOFs): routes to various MOF topologies, morphologies, and composites. Chem. Rev..

[19] Wang Qi, Astruc Didier (2020). State of the art and prospects in Metal−Organic framework (MOF)-Based and MOF-derived nanocatalysis. Chem. Rev..

[20] Baumann A.E., Burns D.A., Liu B., Thoi V.S. (2019). Metal-organic framework functionalization and design strategies for advanced electrochemical energy storage devices. Commun. Chem..

[21] Furukawa H., Cordova K.E., Keeffe M.O., Yagh O.M. (2013). The chemistry and applications of metal-organic frameworks. Science.

[22] Safaei M., Foroughi M.M., Ebrahimpoor N., Jahani S., Omidi A., Khatami M. (2019). A review on metal-organic frameworks: synthesis and applications. Trends Anal. Chem..

[23] Huang W.H., Li X.M., Yang X.F., Zhang X.X., Wang H.H., Wang H. (2021). The recent progress and perspectives on metal- and covalent-organic framework based solid-state electrolytes for lithium-ion batteries. Mater. Chem. Front..

[24] Wei T., Wang Z., Zhang Q., Zhou Y., Sun C., Wang M., Liu Y., Wang S., Yu Z., Qiu X., Xu S., Qin S. (2022). Metal–organic framework-based solid-state electrolytes for all solid-state lithium metal batteries: a review. CrystEngComm.

[25] Wen W., Wang Z., Wang A., Zeng Q., Chen P., Wen X., Li Z., Li Z., Liu W., Zhang L. (2021). A metal–organic framework-5-incorporated all-solid-state composite polymer electrolyte membrane with enhanced performances for high-safety lithium-ion batteries. Energy Technol..

[26] Chu Z., Gao X., Wang C., Wang T., Wang G. (2021). Metal–organic frameworks as separators and electrolytes for lithium–sulfur batteries. J. Mater. Chem. A.

[27] Rodriguez M.U., Vílchez S.V., Olaeta A.M., Luis R.F., Goikolea E., Costa C.M., Mendez S.L., Marijuan A.F., Larramendi I.R. (2022). Exploring ionic liquid-laden metal-organic framework composite materials as hybrid electrolytes in metal (ion) batteries. Front. Chem..

[28] Wu H.B., Lo X.W. (2017). Metal-organic frameworks and their derived materials for electrochemical energy storage and conversion: promises and challenges. Sci. Adv..

[29] Liu Z., Zhang K., Huang G., Xu B., Hong Y., Wu X., Nishiyama Y., Horike S., Zhang G., Kitagawa S. (2022). Highly processable covalent organic framework gel electrolyte enabled by side-chain engineering for lithium-ion batteries. Angew. Chem. Int. Ed..

[30] Zhu F., Bao H., Wu X., Tao Y., Qin C., Su Z., Kang Z. (2019). High-performance metal–organic framework-based single ion conducting solid-state electrolytes for low-temperature lithium metal batteries. ACS Appl. Mater. Interfaces.

[31] Ke F.S., Wu Y.S., Deng H. (2015). Metal-organic frameworks for lithium ion batteries and supercapacitors. J. Solid State Chem..

[32] Fu X., Yu D., Zhou J., Li S., Gao X., Han Y., Qi P., Feng X., Wang B. (2016). Inorganic and organic hybrid solid electrolytes for lithium-ion batteries. CrystEngComm.

[33] Angulakshmi N., Zhou Y., Suriyakumar S., Dhanalakshmi R.B., Satishrajan M., Alwarappan S., Alkordi M.H., Stephan A.M. (2020). Microporous metal − organic framework (MOF)-Based composite polymer electrolyte (CPE) mitigating lithium dendrite formation in all-solid-state-lithium batteries. ACS Omega.

[34] Zhang Z., Huang Y., Li C., Li X. (2021). Metal − organic framework-supported poly(ethylene oxide) composite gel polymer electrolytes for high-performance lithium/sodium metal batteries. ACS Appl. Mater. Interfaces.

[35] Liu Y., Zeng Q., Chen P., Li Z., Chen A., Guan J., Wang A., Zhang L. (2021). Modiﬁed MOF-based composite all-solid-state polymer electrolyte with improved comprehensive performance for dendrite-free Li-ion batteries. Macromol. Chem. Phys..

[36] Wen W., Wang Z., Wang A., Zeng Q., Chen P., Wen X., Li Z., Li Z., Liu W., Zhang L. (2021). A metal–organic framework-5-incorporated all-solid-state composite polymer electrolyte membrane with enhanced performances for high-safety lithium-ion batteries. Energy Technol..

[37] Chen N., Li Y., Dai Y., Qu W., Xing Y., Ye Y., Wen Z., Guo C., Wu F., Chen R. (2019). A Li^+^ conductive metal organic framework electrolyte boosts the high-temperature performance of dendrite-free lithium batteries. J. Mater. Chem. A.

[38] Wu X., Chen K., Yao Z., Hu J., Huang M., Meng J., Ma S., Wu T., Cui Y., Li C. (2021). Metal organic framework reinforced polymer electrolyte with high cation transference number to enable dendrite-free solid state Li metal conversion batteries. J. Power Sources.

[39] Suriyakumar S., Kanagaraj M., Angulakshmi N., Kathiresan M., Nahm K.S., Walkowiak M., Wasínski K., Półrolniczak P., Stephan A.M. (2016). LiFePO_4_ cells with PEO-based composite electrolytes encompassing metal organic frameworks. RSC Adv..

[40] Suriyakumar S., Gopi S., Kathiresana M., Bose S., Gowd E.B., Naird J.R., Angulakshmi N., Meligrana G., Bella F., Gerbald C., Stephan A.M. (2018). Metal organic framework laden poly(ethylene oxide) based composite electrolytes for all-solid-state Li-S and Li-metal polymer batteries. Electrochim. Acta.

[41] Qing X., Li J., Wang Z., Chen M., Lin J., Lin X. (2020). A functionalized metal organic framework-laden nanoporous polymer electrolyte for exceptionally stable lithium electrodeposition. Chem. Commun..

[42] Han Q., Wang S., Jiang Z., Hu X., Wang H. (2020). Framework nanosheets with improved electrochemical stability for all-solid-state Li metal batteries. ACS Appl. Mater. Interfaces.

[43] Wang Z., Wang S., Wang A., Liu X., Chen J., Zeng Q., Zhang L., Liu W., Zhang L. (2018). Covalently-linked metal–organic framework (MOF)-polymer all-solid-state electrolyte membrane for room temperature high performance lithium batteries. J. Mater. Chem. A.

[44] Huo H., Wu B., Zhang T., Zheng X., Ge L., Xu T., Guo X., Sun X. (2019). Anion-immobilized polymer electrolyte achieved by cationic metal-organic framework ﬁller for dendrite-free solid-state batteries. Energy Storage Mater..

[45] Sun C.C., Yusuf A., Li S.W., Qi X.L., Ma Y., Wang D.Y. (2021). Metal organic frameworks enabled rational design of multifunctional PEO-based solid polymer electrolytes. Chem. Eng. J..

[46] Wu J.F., Guo X. (2019). MOF-derived nanoporous multifunctional ﬁllers enhancing the performances of polymer electrolytes for solid-state lithium batteries. J. Mater. Chem. A.

[47] Zhang Z., Huang Y., Gao H., Hang J., Li C., Liu P. (2020). MOF-derived ionic conductor enhancing polymer electrolytes with superior electrochemical performances for all solid lithium metal batteries. J. Membr. Sci..

[48] Zhang Z., Tian L., Zhang H., Xu H., Dong P., Zhang Y., Long D. (2022). Hexagonal rodlike Cu-MOF-74-Derived filler-reinforced composite polymer electrolyte for high-performance solid-state lithium batteries. ACS Appl. Energy Mater..

[49] Bai S., Sun Y., Yi J., He Y., Qiao Y., Zhou H. (2018). High-power Li-metal anode enabled by metal-organic framework modiﬁed electrolyte. Joule.

[50] Majid M.F., Zaid H.F.M., Kait C.F., Ahmad A., Jumbri K. (2022). Ionic Liquid@Metal-organic framework as a solid electrolyte in a lithium-ion battery: current performance and perspective at molecular level. Nanomaterials.

[51] Sicolo S., Kalcher C., Sedlmaier S.J., Janek J., Albe K. (2018). Diffusion mechanism in the superionic conductor Li_4_PS_4_I studied by first-principles calculations. Solid State Ionics.

[52] Deng Z., Radhakrishnan B., Ong S.P. (2015). Rational composition optimization of the lithium-rich Li_3_OC_11_–xBr_x_ anti-perovskite superionic conductors. Chem. Mater..

[53] Jeong K., Park S., Jung G.Y., Kim S.H., Lee Y.H., Kwak S.K., Lee S.Y. (2019). Solvent-Free, single lithium-ion conducting covalent organic frameworks. J. Am. Chem. Soc..

[54] Bai S., Sun Y., Yi J., He Y., Qiao Y., Zhou H. (2018). High-power Li-metal anode enabled by metal-organic framework modified electrolyte. Joule.

[55] Xia Y., Xu N., Du L., Cheng Y., Lei S., Li S., Liao X., Shi W., Xu L., Mai L. (2020). Rational design of ion transport paths at the interface of Metal−Organic framework modified solid electrolyte. ACS Appl. Mater. Interfaces.

